# Supplementary material for: Development of a collaborative chronic care model for management of cardiometabolic disease in low- and middle-income countries
Source: PLoS One. 2026 Mar 16;21(3):e0344527. doi: 10.1371/journal.pone.0344527 (PMC12991268; doi:10.1371/journal.pone.0344527)
Supplement: Attachment — Submitted filename: Response to Reviewer.docx [file pone.0344527.s001.docx]

**Response to Reviewer Comments**

**Reviewer #1:** The manuscript provides a timely and relevant overview of cardiometabolic diseases (CMDs), particularly highlighting the disproportionate burden borne by low- and middle-income countries (LMICs). The authors propose an adaptation of the Chronic Care Model (CCM) as a potential response, with an emphasis on person-centred and community-orientated care. A key strength of the study lies in its effort to triangulate multiple sources of data (from a literature review to community engagement), which are then synthesised into a proposed model. The emphasis on preserving core CCM principles while adapting the model to LMIC contexts is commendable.

**Reviewer comment #1:** To further strengthen the manuscript, it may be helpful to situate this work more clearly within the broader body of literature that has sought to revise or adapt the traditional CCM. Several recent iterations have moved toward greater collaboration and integration across sectors and referencing these could help clarify how the proposed Collaborative Chronic Care Model (CoCCM) builds upon, or diverges from, existing efforts.

**Author response:**

We thank the reviewer for this valuable suggestion. The literature review section has been updated to include a detailed discussion of adaptations made to the traditional Chronic Care Model (CCM), highlighting how our proposed CoCCM builds on existing revisions**. (Pages 8–9 of the manuscript).**

**Adaptations to the traditional chronic care model**

Recent literature highlights substantial adaptations to the traditional CCM, aimed at strengthening cross-sector collaboration and integration in chronic disease management. These revisions emphasize multidisciplinary teamwork, leveraging technology, systemic reforms, and patient-centered and holistic care, ensuring a more holistic and coordinated approach.

Multidisciplinary teams (MDTs) integration now forms the backbone of the revised CCM, incorporating pharmacists, nurses, general practitioners, and psychologists to deliver comprehensive, patient-centered care (1,2). The inclusion of pharmacists improves medication adherence and reduces errors, enhancing outcomes while reducing healthcare costs (1). The integration of digital health tools, eHealth platforms, and interoperable information systems strengthens communication and data sharing across sectors (2,3). Digital solutions enhance coordination of patient care by enabling proactive monitoring, population-level data analysis, and have also been critical in the integration of mental and physical health management (4). The eHealth Enhanced CCM (eCCM) highlights the value of virtual communities and feedback loops in boosting patient engagement (5).

Recent literature calls for policy reforms, shared care plans, and interprofessional training to standardize collaboration and clarify roles and responsibilities within MDTs (2). Revisions of the CCM include integration of physical, emotional, and social dimensions of health, hence fostering individualized care plans that improve satisfaction and outcomes. The Expanded Chronic Care Patient–Professional Partnership Model (E2C3PM) is a novel integrated model that leverages patient experience and knowledge, mediation, therapeutic education, and partnership to empower patients, rebalance power dynamics, and foster a more comprehensive and collaborative approach to chronic disease management, leading to improved care integration and outcomes (6). Community integration across healthcare and social sectors improves care coordination for complex patient needs, reducing exclusion and supporting continuity of care. Applications of the CCM in contexts such as the Veteran’s Health and correctional systems demonstrate its adaptability to diverse populations and institutional settings (7). Community-based organizations and primary care nurses are increasingly recognized as key actors in supporting self-management and extending care beyond clinical settings (8).

Despite these advancements, barriers such as resistance to change, lack of standardized protocols, and limited adoption of eHealth and patient-centred and partnership approaches persist (3). Effective implementation requires sustained innovation, organizational cultural shifts, and adaptation to local contexts to fully optimize chronic care delivery.

**Reviewer comment #2.** While the focus on CMDs is well-argued, it would be valuable to more explicitly demonstrate how the CoCCM offers unique utility for CMD management compared to its application for other chronic conditions. Clarifying this would enhance the specificity and relevance of the proposed model.

Apart from its use in management cardiometabolic diseases, how can we explicitly demonstrate the application of the Collaborative Chronic Care Model (CoCCM) for other chronic conditions.

**Author response:**

We agree with this comment and have elaborated in the discussion section **(page30)** how the CoCCM is specifically suited for cardiometabolic disease (CMD) management, in comparison to other chronic medical conditions.

The CoCCM is particularly well-suited for managing chronic diseases due to its comprehensive, patient-centered approach that integrates various aspects of healthcare delivery. CMDs are the commonest clusters of multiple long-term conditions. This model addresses the unique challenges of CMD by emphasizing coordinated care, patient self-management, and the use of evidence-based guidelines, which are crucial for managing the complexities associated with CMD. The CoCCM's design facilitates improved patient outcomes and resource utilization, making it an effective framework for CMD management.

The CoCCM has been applied to a variety of chronic conditions, including mental health disorders, chronic respiratory diseases, chronic kidney disease, and conditions affecting the elderly(9). In the management of cardiometabolic disease, the CoCCM offers an effective framework for managing cardiometabolic diseases through its integrated, team-based, and patient-centered approach. It addresses the complex and interrelated nature of conditions such as cardiovascular disease, type 2 diabetes, and obesity by promoting coordination among healthcare providers and emphasizing evidence-based, patient-focused care(10).

Key components of the CoCCM include team-based care, where multidisciplinary professionals collaboratively manage both metabolic and cardiovascular aspects of disease at the different levels of care (11,12). The patient-centered approach enhances self-management and engagement, enabling patients to take an active role in decision making for their care(13). The use of evidence-based guidelines ensures optimal treatment, to reduce cardiovascular risks (11). Integrated care delivery improves continuity and coordination across healthcare settings, hence mitigating fragmentation of care (12,14). The model’s focus on prevention and early intervention supports timely identification and management of risk factors, reducing the onset of complications.

**Reviewer comment #3.** There appears to be some misalignment between the stated knowledge gap, namely, the lack of evidence around the implementation and scale-up of collaborative care models in LMICs, and the study’s stated aim to develop a person-centred model of care for CMD management. Clarifying how the study responds to this gap may help improve the coherence of the paper.

**Author response:**

We clarified the knowledge gap on **Page 10**, emphasizing that our study responds to the lack of evidence on adaptation, scalability, and sustainability of collaborative care models in LMICs

There exists knowledge gap in the adaptation, scalability and sustainability of chronic care models that have been successfully implemented to fit the LMIC context in relation to constraints related to the health system, infrastructure and social and cultural factors. To date, evidence on the implementation of collaborative care models emanate from high income countries with little adaptation to LMIC settings (15–17). There is therefore a lack of evidence on how to effectively adapt and scale interventions in diverse LMIC settings, which is crucial for addressing common chronic conditions such as cardiometabolic diseases and mental health (9,16). Many LMICs face health system constraints such as inadequate infrastructure, shortage of trained health professionals, limited access to essential medications and diagnostic tools (18), which may pose significant challenges in the implementation of collaborative care models. This calls for the need to develop and test novel low-cost approaches which are tailored to resource-limited settings to address these disparities (18). There is also knowledge gap in the understanding the use of digital technology, health care provider training, and supervisory structures including how to effectively integrate mid-level practitioners and community health workers necessary for effective chronic care management (19).

**Reviewer comment #4.** The framing of LMICs (and Africa in particular) could be strengthened. At times, the manuscript presents a high-income versus low-income dichotomy, which may unintentionally reinforce deficit-based narratives. It also overlooks evidence showing that cardiovascular disease (CVD) mortality has plateaued, or even increased, in some high-income countries.

**Author response:**

Thank you for this comment. We have provided a reference stating the increase and plateau of CVD in high income countries.

This has been noted on **Page 4** of the manuscript.

However, data published in the World Health Organization Mortality Database indicate that the rate of decline in mortality due to cardiovascular disease in some high-income countries has slowed down or plateaued particularly for persons aged 35-74yrs; for example, North America (USA and Canada), mortality rates due to CVD have increased in recent years (20). In Switzerland, while CVD mortality continues to decrease in most of the population, specific age groups, such as those aged 60-74, have seen a plateau in stroke and coronary heart disease mortality rates. The plateau could be attributed to several factors such as lack of regular physical activity, unhealthy diets, high alcohol consumption, persistent smoking and social inequalities among different segments of the population (21).

**Reviewer comment #5.** A more balanced approach would be to highlight both the ongoing challenges, the local innovations and gains in CMD management seen in specific African countries such as Kenya, Ghana, and Mozambique (which the paper later references). This would offer a more nuanced and empowering perspective, rather than generalising across the entire African continent or all LMICs.

**Author response:**

On-going challenges and local innovation has been clarified on **pages 10-11** of the manuscript.

**Challenges and innovations in CMD management**

Management of CMD in Kenya, Ghana and Mozambique face challenges which include health system factors, social and cultural factors such as lack of access to early diagnostic tools, lack of access to essential medicines and frequent stockouts in public hospitals, use of traditional medicine and disproportionate distribution of health care providers between urban and rural settings. For instance, Kenya has reported an increase in the prevalence of hypertension, but only 4% of the patients under treatment achieve control(22). With regards to early detection of diabetes, more that 88% of Kenyans have never had their blood glucose tested, leading to late diagnosis (22). Less than 50% of hospitals in Kenya have the equipment and trained staff required for essential diagnostic procedures like electrocardiography and ultrasonography and only 49% of hospitals have a complete stock of necessary medications, with frequent stock-outs reported (18). Similarly in Ghana challenges include inadequate infrastructure, workforce shortages, fragmented coordination of care, high costs of care and out-of-pocket expenditure, and governance inefficiencies for chronic disease management, reliance on traditional medicine, cultural beliefs and delayed care seeking (23,24)

Promising innovations in the management of CMDs in Kenya, Ghana and Mozambique include community-based lifestyle interventions which have reported significant reductions in metabolic syndrome markers such as improved diet and exercise adherence(25), and task-sharing models focusing on community health worker-led screening and linkage programs leveraging on digital technology (26). The *Akoma Pa* digital, faith-based pilot intervention tested in Ghana has reported higher patient follow-up rates, improved blood pressure and glycemic control, and demonstrated favorable cost-effectiveness(27). Mozambique has adapted community-based medication delivery models from successful HIV care programs to improve chronic disease management (28).

**Reviewer comment #6.** It may also be helpful to reconsider the use of the term “sub-Saharan Africa”, as it has been critiqued in scholarly literature for its lack of specificity and its problematic historical associations.

**Author response:**

This is correct.

The use of the term sub-Saharan Africa throughout the manuscript has been reviewed since this also excludes North Africa from the rest of the continent. Where reference is made to the three study countries (Kenya, Ghana and Mozambique) this has been specified to refer to the three countries, but where existing literature focus is on SSA, this has been maintained.

**Reviewer comment #7.** The manuscript could benefit from more explicit articulation of how the proposed CoCCM was shaped by the specific needs, characteristics, and resources of people living with CMD in the LMIC (or African) context. While the literature review and data collection are comprehensive, it is not entirely clear what was learned about the unique needs, characteristics and resources across the study settings and how these inputs were translated into design elements of the model. More detail on this process would enhance the model’s contextual relevance and practical applicability.

**Author response:**

Thank you for this comment.

We have expanded the manuscript to outline how the proposed CoCCM was shaped by the specific needs, characteristics, and resources of people living with CMD in the LMIC (or African) context.

This is included on **pages 11-12** of the manuscript

The qualitative interviews and stakeholder workshops revealed several key unmet needs among people living with cardiometabolic diseases (CMDs). These included low awareness of disease signs and symptoms, resulting in poor health-seeking behaviour and late diagnosis. Patients also cited limited access to essential medicines, high costs of laboratory monitoring, and unhealthy dietary practices. Care-seeking was often initiated by an acute episode such as collapse. Many patients wanted to treat CMDs as acute illnesses and often asked if they could discontinue taking medication if they got symptom relief. Additionally, there was a notable lack of health education and suitable educational materials to support disease management and awareness. These perceptions informed the design of the SME and the community linkages components of the intervention. With regards to available resources, there were existing national policies and guidelines in the three countries which provided a framework for intervention design. However, there were notable gaps in the implementation and referral practices. Stakeholders also cited negative health care provider attitude, provision of fragmented care and the need to train healthcare providers to improve communication among themselves and with patients. At the community level, the following community structures were identified for intervention delivery: patient support groups, community health workers and volunteers, community leaders, women groups, church groups and keep-fit clubs.

With regards to intervention delivery stakeholders and persons with lived experiences noted that the intervention should be designed in such a way that it is delivered at certain venues, days, times to address challenges around transport and work-life commitments. It also transpired that literacy was a unique need across the study settings – this prompted the development team to develop participant resources including patient facing materials that were user-friendly and visually-led (less text-heavy) and culturally relevant. This key information was fed back to the development team and was considered when developing the intervention and the Theory of Change.

**Reviewer comment #8.** Finally, by focusing mainly on individual behaviour change as the key aspect of the CoCM model, the authors risk advocating for a care model that ignores how structural factors constrain or facilitate individual agency.

**Author response:**

We thank the reviewer for this important observation. We agree that focusing solely on individual behaviour risks underrepresenting the broader contextual and structural factors that influence care delivery and patient outcomes. While our development work emphasised behavioural change at the individual level as a key entry point, the intervention was conceptualised as a complex, multi-component model rather than a single-level behavioural approach.

In addition to self-management support, which was the primary focus of this phase, the model incorporated elements of delivery system design, to promote proactive, team-based care, and community linkages, to strengthen connections with local resources and address wider social determinants of health. These components were intended to provide structural and environmental supports that facilitate, rather than isolate, individual behaviour change.

We acknowledge that some components (e.g., decision support and clinical information systems) were not consistently implemented across sites and therefore were not the focus of this analysis. This has been clarified in the limitations section, where we also note that future work should more explicitly examine how contextual and structural factors influence the sustainability and scalability of the model. – see page 30 of the manuscript

**Reviewer #2:**

This manuscript presents the development of a health care model for people with multiple chronic cardiometabolic diseases (CMD/MLTC) in sub-Saharan Africa. The authors clearly articulate the study’s aim, and ethical clearance was appropriately obtained from the relevant committees in Kenya, Ghana, and Mozambique.

The development of the model was informed by a systematic review, qualitative synthesis, and a needs assessment with stakeholders. The resulting CREATE intervention comprises five key components: a self-management education (SME) programme, a training package for educators to deliver the SME, a training package to upscale health care providers and community champions in CMD, a decision making tool for a data registry, and an improved referral pathway.

**Reviewer comment #1.** Although the authors have stated that all information is included in the manuscript and the supporting information files, I could not find any access links to the supporting files. I believe that resources such as guides for the SME sessions, community linkage training materials, and shared decision-making guidelines, are of interest in the context of this manuscript. Inclusion of those resources in the supporting information files should be considered.

**Author response:**

For the purpose of this manuscript, we present in detail the SME objectives, features and content and BCTs. Due to IP and copyright of the intervention resources, we are unable to upload the full resources and training guides. We have however, in response to the reviewer’s comment, uploaded images of example intervention resources to provide context to the reader – file name **“CREATE resources”**

We have also included a link to the health care providers training which is publicly available on Youtube. The aim of the health care providers training was to educate and empower clinicians on consultation techniques during chronic disease consultations. Here is the link: <https://www.youtube.com/watch?v=7ho7LS8URGQ>

**Reviewer comment #2.** The manuscript is generally well-structured and linguistically sound.

**Author response:**

Thank you for this comment. This is much appreciated

**Reviewer comment #3.** The following **minor revisions** are recommended to accept the manuscript for publication. All cited pages below refer to the manuscript page at the bottom of each page and not the pdf page.

**Author response:**

The following minor comments have been made on the manuscript.

**Reviewer comment #4.** 1. To my understanding, the chronic conditions focused on by this program are T2DM, hypertension, cholesterol, and cardiovascular disease. I suggest describing in the manuscript why the model was limited to these four chronic conditions.

**Author response:**

This is an important comment on the need to justify the focus on the T2DM, hypertension, cholesterol, and cardiovascular disease.

This is described on **pages 11-12** of the manuscript.

Our decision to focus on these four conditions was informed by evidence showing an increase in the cardiovascular morbidity and mortality in LMICs, the need to improve patient outcomes, and our clinical knowledge and global research expertise. These are also the commonest cardiometabolic clusters of Multiple Long-Term Conditions (MLTCs). Between 1990 and 2019, there has been a 131.7% increase in CVD prevalence (29) which compounds cardiometabolic disease burden as it increases all-cause mortality risk by 4-7 fold (30). Cardiometabolic conditions often co-exist in an individual making an integrated care approach more efficient.

There is an interconnectedness of these four chronic conditions which exacerbates morbidity and mortality, making it crucial to address them collectively. These conditions are prevalent and contribute to the high morbidity and mortality in these countries. Type 2 diabetes, hypertension and dyslipidemia are significant risk factors for cardiovascular disease, but prevention and management are suboptimal due to inadequate healthcare systems and lack of resources. For example, hypertension remains the major risk factor for stroke and hence also a major contributor to the onset of CVD (31). Persons living with type 2 diabetes are 2-3 times more likely to experience cardiovascular events (32). In addition, hypertension is twice as common among people with diabetes, and up to 75% of cardiovascular disease in diabetes attributable to hypertension (33).

**Reviewer comment #5.** Table 3: The alignment between SME features and SME sessions in Table 3 is unclear. For example, SME feature "Focus on creating lifestyle habits that are tailored to people's routine" is in the same row as SME sessions "Recommendations of moderate vs vigorous", which seem unrelated to each other. I suggest modifying the data presentation to avoid misunderstanding. Some options: Option 1. nested table (instead of aligning features and sessions row by row, nest the features and sessions under the same objective: one row per objective, list all features in the objective in the same row in column 2, list all sessions under the objective in the same row in column 3). Option 2. bulleted concept map (diagram). Other forms may be utilized according to how the authors see fit.

**Author response:**

We have made the revisions on the format of Table 3 - see **page 22-24** of the manuscript

**Table 3:** CREATE SME education design objectives, features and sessions content.

| SME design objectives | SME features | SME sessions |
| --- | --- | --- |
| To raise awareness on the importance of clinical medicine to address the cultural/ individual belief system | Trusted and credible sources, evidence-based work developed by credible medical teams locally, or internationally (regarding T2D and CVD).  Share examples of personal lifestyle changes (based on clinical medicine and behaviour change).  Local educator will present information on the current healthcare system and possible opportunities of free medication (educator note).  Local educator will be trained to acknowledge and address queries raised regarding traditional medicine. | T2D and Heart Story  Medication myths Activity (card game of individual quotes |
| To encourage healthy eating through a reduced salt, fat and carbohydrate diet? | Emphasis on affordable food providing examples of food relevant to the target audience.  Focus on creating lifestyle habits that rely less on carb-heavy, high salt and fat-based meals and lifestyle changes to traditional meals.  Focus on creating lifestyle habits that rely more on high fibre-based meals.  Build on self-efficacy and skills (link with Social Cognitive Theory). | Food activity game: Activity for educator to source local food and food packaging to bring to the session (Fat, sugar, salt).  Food Table (example of culturally relevant foods)  How to calculate the level of grams in the food portion (using spoons, instead of sugar cubes).  Sort the Fat.  100 calorie game (walk it off).  Fibre activity. |
| To help people understand the importance of high intensity exercise? | Acknowledge low intensity exercise but emphasise on high intensity physical activity. Provide examples on images.  Focus on creating lifestyle habits that are tailored to people’s routine. | Physical activity continuum  Recommendations of moderate vs vigorous physical activity.  Provide examples of physical activity (e.g. walking with friend, skipping, playing football). |
| To educate about CMD and CMD management | Focus less on text, and more on visuals to present information about CMD.  Provide very brief health messages using simple language. | Participant handbook mainly images.  Description of CMD displayed with images.  For the participant story, use images or drawing. |
| To promote user autonomy | Provide an environment for people to choose their own behaviour change, goals, action plan.  Provide an environment for people to reflect on questions (not expect the facilitator to be the expert).  Promote involvement and engagement in interactive activities and group discussions  Promote the ethos of the programme that people are responsible for their own behaviour. | Self-management plan and provide time to reflect and complete.  Highlight the ethos and approach in the educator’s training, and guided throughout the curriculum.  Set scene at the beginning of the session (e.g. interactive learning) |
| To promote intrinsic motivation and stress management | Use positive language throughout the programme and use key facilitation skills (listening, acknowledging people’s stories, enable group discussion). | Relaxation square activity |
| To promote engagement | Consider the time and location of the training programme.  Consider the cost to attend the programme.  Promote SMES on radio and other media. |  |

**Reviewer comment #6**. Kindly specify who the audience for the SME program was: HCP, patients, or both. (**page 22** of the manuscript)

**Author response:**

The audience of the SME program were patients however health care providers were trained using the CREATE curriculum on how to deliver the SME programme to the patients.

**Reviewer comment #7**. The six components of CREATE CoCCM in the article were 1) Self-Management support, 2) Clinical information system, 3) Delivery system design (streamlining the referral pathway), 4) Organisation of health care, 5) Decision support (which included health care provider training), and 6) Community linkages; these do not match the components in the results section of the Abstract. Consider streamlining for clarity.

**Author response:**

Thank you for this comment and the need for clarification. We have revised the abstract to reflect the six intervention components.

1) Self-Management support, 2) Decision support (which included health care provider training), 3) Community linkages, 4) Organisation of health care, 5) Clinical information system, and 6) Delivery system design (streamlining the referral pathway).

**Reviewer comment #7**. Please check the manuscript and amend some grammatical and typographical errors, including, but not limited to:
- consistency with American or British spelling (British spelling was used for the phrase "Randomised Control Trials", whereas other parts of the manuscript uses American English).
- inaccurate use of capital letters where they are not needed e.g. "Randomised Control Trials" on page 8 and "Systolic Blood Pressure" on page 25.
- consistency with abbreviations; some terms' abbreviations have been specified but unabbreviated terms are used again in the later parts of the manuscript. For example, "Multiple long-term conditions (MLTC)" has been cited on page 8, but is inconsistently written as the unabbreviated form (pages 8, 9, 10) and abbreviated form (page 24). Similarly for "Chronic Care Model (CCM)" and "Collaborative care models".
- grammatical errors such as "traditional high calorific calories" -> "high calorific value" or "high calorie foods".

**Author response:**

Thank for the comments above, these grammatical revisions have been made throughout the manuscript.

**References**

1.	Alnahidh AAA, Alanazi SMI, Alghamdi ASA, Alblawi S, Al-Koikibi ASS, Shikhani FMH, et al. The Integral Role of Multidisciplinary Teams in the Management of Chronic Illnesses: Enhancing Patient Outcomes through Collaborative Care Models. Journal of Ecohumanism. 2024 Dec 30;3(8).

2.	Alanazi AZM, Abdulrahman MA, Alsaiyegh BMH, Alabdullatif WIA, Buhelal RH, Almutairi FS, et al. Collaborative Chronic Disease Management in Primary and Emergency Care: A Multidisciplinary Approach Integrating Health Administration, Pharmacy, Nursing, General Practice, EMS, Psychology, and Laboratory Services. Journal of Ecohumanism. 2024 Dec 29;3(8).

3.	Raharjo TP, Purnomo ID, Tlonaen AW, Taurensa V, Petrahaurissa HY, Indrianto A, et al. THE EVOLUTION AND IMPACT OF COLLABORATIVE LEADERSHIP ACROSS SECTORS: A SYSTEMATIC REVIEW OF RECENT EVIDENCE (2019-2024). Journal of Scientech Research and Development. 2024 Nov 7;6(2):138–46.

4.	Sullivan WP, Wahler EA. Chronic care, integrated care, and mental health: Moving the needle now. Soc Work Ment Health. 2017 Nov 2;15(6):601–14.

5.	Gee PM, Greenwood DA, Paterniti DA, Ward D, Miller LMS. The eHealth Enhanced Chronic Care Model: A Theory Derivation Approach. J Med Internet Res. 2015 Apr 1;17(4):e86.

6.	Pomey M, Schaad B, Lasserre‐Moutet A, Böhme P, Jackson M. Towards a New Integrated Model for Taking Into Account the Experiential Knowledge of People With Chronic Diseases, Integrating Mediation, Therapeutic Education and Partnership: The Expanded Chronic Care Patient–Professional Partnership Model. Health Expectations. 2024 Oct 7;27(5).

7.	Kim B, Bolton RE, Hyde J, Fincke BG, Drainoni ML, Petrakis BA, et al. Coordinating across correctional, community, and VA systems: applying the Collaborative Chronic Care Model to post-incarceration healthcare and reentry support for veterans with mental health and substance use disorders. Health Justice. 2019 Dec 12;7(1):18.

8.	Ratner SP, DeWalt DA. Future Directions in Chronic Illness Care. In: Chronic Illness Care. Cham: Springer International Publishing; 2018. p. 549–55.

9.	Chang LS, Vaduganathan M, Plutzky J, Aroda VR. Bridging the Gap for Patients with Diabetes and Cardiovascular Disease Through Cardiometabolic Collaboration. Curr Diab Rep. 2019 Dec 4;19(12):157.

10.	Reiter-Brennan C, Dzaye O, Davis D, Blaha M, Eckel RH. Comprehensive Care Models for Cardiometabolic Disease. Curr Cardiol Rep. 2021 Mar 24;23(3):22.

11.	Ahmed MDF, Jaman A, Barman M, Iloanusi C, Muna MK, Arefine R. Advancing Patient Outcomes through Collaborative Care: Evidence from Integrated Behavioral Healthcare. Europasian Journal of Medical Sciences. 2024 Dec 31;6.

12.	Alaujan BS, Zamzami BA, Ababtain HA, Altalouhi FM, Alshammari MH, Alenizi ES, et al. Integrating preventive cardiology in primary care. Int J Health Sci (Qassim). 2021 Jan 15;5(S1):1049–66.

13.	Whitfield J, Owens S, Bhat A, Felker B, Jewell T, Chwastiak L. Successful ingredients of effective Collaborative Care programs in low- and middle-income countries: A rapid review. Cambridge Prisms: Global Mental Health. 2023 Mar 17;10:e11.

14.	Haregu TN, Mahat K, Miller SM, Oldenburg B. Improving diabetes prevention and management amidst varied resources: from local implementation to global learnings. Transl Behav Med. 2020 Feb 3;10(1):1–4.

15.	Donohue JF, Elborn JS, Lansberg P, Javed A, Tesfaye S, Rugo H, et al. Bridging the “Know-Do” Gaps in Five Non-Communicable Diseases Using a Common Framework Driven by Implementation Science. J Healthc Leadersh. 2023 Jul;Volume 15:103–19.

16.	Acharya B, Ekstrand M, Rimal P, Ali MK, Swar S, Srinivasan K, et al. Collaborative Care for Mental Health in Low- and Middle-Income Countries: A WHO Health Systems Framework Assessment of Three Programs. Psychiatric Services. 2017 Sep 1;68(9):870–2.

17.	Ribeiro AL, Otto CM. Heartbeat: Implementation gaps in cardiovascular care in middle–low income countries. Heart. 2017 Dec 15;103(23):1837–9.

18.	Schwarz D, Dhungana S, Kumar A, Acharya B, Agrawal P, Aryal A, et al. An integrated intervention for chronic care management in rural Nepal: protocol of a type 2 hybrid effectiveness-implementation study. Trials. 2020 Dec 29;21(1):119.

19.	Lopez AD, Adair T. Is the long-term decline in cardiovascular-disease mortality in high-income countries over? Evidence from national vital statistics. Int J Epidemiol. 2019 Dec 1;48(6):1815–23.

20.	Sorrentino L, Chiolero A, Carmeli C. Cardiovascular mortality trends in Switzerland 1995–2018. Eur J Public Health. 2022 Nov 29;32(6):891–3.

21.	KNBS. Kenya - Kenya Stepwise Survey for Non Communicable Diseases Risk Factors 2015. Nairobi; 2015.

22.	Abdul-Samed AB, Jahan Y, Reichenberger V, Peprah EB, Agyekum MP, Lawson H, et al. Barriers and facilitators of primary care management of type II diabetes mellitus in the West African sub-region: A scoping review. PLOS Global Public Health. 2025 May 5;5(5):e0003733.

23.	Boima V, Doku A, Hayfron-Benjamin C, Twumasi AAA, Awuah RB, Selom JE, et al. Level of understanding and community-level barriers to the management of hypertension: a qualitative study in eight coastal communities in Ghana. BMJ Glob Health. 2025 Aug 26;10(8):e017511.

24.	Okube OT, Kimani S, Mirie W. Community-based lifestyle intervention improves metabolic syndrome and related markers among Kenyan adults. J Diabetes Metab Disord. 2022 Jun 21;21(1):607–21.

25.	Hinneh T, Mensah B, Kwanin C, Okonkwo C, Byiringiro S. Digital health tools in hypertension management in sub-Saharan Africa: a scoping review of barriers and facilitators of adoption into mainstream healthcare. BMJ Health Care Inform. 2025 Oct 10;32(1):e101261.

26.	Owusu IK, Adomako-Boateng F, Kueffer F, Guy M, Lang at C, Grossman DB, et al. Novel Hypertension Management Model of Care Improves Blood Pressure Control in a West African Population. J Hypertens (Los Angel). 2018;07(03).

27.	Queiroz IG de, Santos RCM dos, Freitas FMN de O, Figueiredo RS. O IMPACTO DA NUTRIÇÃO NO TRATAMENTO E PREVENÇÃO DE DOENÇAS CRÔNICAS NÃO TRANSMISSÍVEIS: HIPERTENSÃO E DIABETES MELLITUS. Revista ft. 2024 Oct 29;28(139):04–5.

28.	Alhuneafat L, Ta’ani O Al, Tarawneh T, ElHamdani A, Al-Adayleh R, Al-Ajlouni Y, et al. Burden of cardiovascular disease in Sub-Saharan Africa, 1990–2019: An analysis of the Global Burden of Disease Study. Curr Probl Cardiol. 2024 Jun;49(6):102557.

29.	Thienemann F, Ntusi NAB, Battegay E, Mueller BU, Cheetham M. Multimorbidity and cardiovascular disease: a perspective on low- and middle-income countries. Cardiovasc Diagn Ther. 2020 Apr;10(2):376–85.

30.	Moran AE, Roth GA, Narula J, Mensah GA. 1990-2010 Global Cardiovascular Disease Atlas. Glob Heart. 2014 Mar 1;9(1):3.

31.	Roman G, Pantea Stoian A. Cardiovascular Risk/Disease in Type 2 Diabetes Mellitus. In: Type 2 Diabetes - From Pathophysiology to Cyber Systems. IntechOpen; 2021.

32.	Sowers JR, Epstein M, Frohlich ED. Diabetes, Hypertension, and Cardiovascular Disease. Hypertension. 2001 Apr;37(4):1053–9.
